# Pheromone-Based Mating Disruption of *Conogethes punctiferalis* (Lepidoptera: Crambidae) in Chestnut Orchards

**DOI:** 10.3390/insects15060445

**Published:** 2024-06-12

**Authors:** Junheon Kim, Seongchae Jung, Young Un Kim

**Affiliations:** 1 Forest Entomology and Pathology Division, National Institute of Forest Science, Seoul 02455, Republic of Korea; 2 AD Corporation, Andong 36729, Republic of Korea; catidel@daum.net (S.J.); yukim125@naver.com (Y.U.K.)

**Keywords:** *Castanea crenata*, *Conogethes punctiferalis*, (E)-10-hexadecenal, *Cydia kurokoi*

## Abstract

**Simple Summary:**

The study assessed a pheromone-based mating disruption (MD) for the control of *Conogethes punctiferalis* in Korean chestnut orchards. Over two years, field trials showed that MD effectively reduced male *C. punctiferalis* numbers, with male catch inhibition (MCI) ranging from 70.5% to 95.1%. MD treatments, including single-dosage (TS) and double-concentration (TD) or two-application (TT) methods, significantly reduced fruit damage. In 2022, TS achieved 63.9–73.6% efficacy, while in 2023, TD and TT showed variable but notable reductions in pest numbers and fruit damage. The study confirms the MD’s efficacy in mitigating *C. punctiferalis* damage in chestnut orchards, highlighting its potential as an eco-friendly pest management strategy in agroforestry systems.

**Abstract:**

Chestnuts (*Castanea crenata* Siebold and Zucc.) are one of the major agroforestry products in Korea, and *Conogethes punctiferalis* is a major pest of the chestnut fruit. Pheromone-based mating disruption (MD) has emerged as a promising eco-friendly approach to reduce population levels and ultimately mitigate fruit damage. Field trials were conducted over two years (2022–2023) in two commercial chestnut orchards in Central Korea that were infested with *C. punctiferalis*. Compared with the control treatment, the MD treatment effectively reduced the number of male *C. punctiferalis* captured in the MD treatment plots. Male catch inhibition (MCI) rates ranged from 70.5% to 82.7% in 2022 and from 87.8% to 95.1% in 2023. The MD efficacy (%) was calculated based on the total number of chestnut fruits collected and the number of fruits damaged by *C. punctiferalis*. In 2022, the MD efficacy of the single-dosage treatment (TS, 50 g/ha) was 63.9% in Orchard A and 73.6% in Orchard B. In 2023, the MD efficacies of the double-dosage treatment (TD, 100 g/ha) and the two-application treatment (TT, 50 g/ha in June and August) were 60.2% and 77.9% in Orchard A and 50.9% and 64.8% in Orchard B, respectively. This study confirms the efficacy of pheromone-based MD in reducing the *C. punctiferalis* numbers in chestnut orchards and damage to chestnut fruits.

## 1. Introduction

*Castanea crenata* Siebold and Zucc. (Fagales: Fagaceae) is a species of chestnut native to Korea and Japan and is mainly distributed in Korea, Japan, and Northeastern China [1,2]. Chestnuts are one of the major agroforestry products in Korea, with a cultivated area of approximately 15,175 ha producing 13,220 tons annually, amounting to exports of more than $15,148/year [3,4]. However, due to climate change, a shortage of the agricultural working population, and damage from insect pests, chestnut production continues to decline. As insect pests of chestnuts, the yellow peach moth *Conogethes punctiferalis* Guenée (Lepidoptera: Crambidae) (formerly known as *Dichocrocis punctiferalis* (Lepidoptera: Pyralidae)) and chestnut weevil *Curculio sikkimensis* Heller (Coleoptera: Curculionidae) are notorious. *Conogethes punctiferalis* is widely distributed in South and East Asia, Australia, and Papua New Guinea, is often included in the quarantine pest list of many countries in Europe, and is a regulated pest in North America [5,6,7,8]. Larvae are typically polyphagous and can feed on a broad range of hosts in addition to chestnuts, such as peaches, plums, apricots, pears, walnuts, and guavas [9,10,11].

Chemical insecticides are typically used to control the insect pests of chestnuts. Although pesticides can control pests and improve the yield and quality of produce, there are serious concerns about the adverse impacts it might have on the ecosystem, such as the occurrence of resistant strains, the reduction in the amount of natural enemies and pollinator insects, and the risk of residues and exposure to farmers [12,13,14]. To overcome such problems, new alternative methods are needed. Among the many alternative methods, pheromone-based mating disruption (MD) technology provides a good alternative, avoiding the drawbacks associated with chemical insecticides [15,16]. The use of MD has proven to be a successful method for controlling various pests, including forest pests, among others, affecting more than 770,000 ha [17].

Among the two major pests of chestnut, only the pheromone of *C. punctiferalis* has been identified. Two female-produced sex pheromones for *C. punctiferalis* were identified in Japan as (*E*)-10-hexadecenal (E10-16:Ald) and (*Z*)-10-hexadecenal (Z10-16:Ald), for which a mixture with a ratio of 90:10 of the *E* to *Z* isomers was the most efficient for attracting males of this species [18]. In China, the most attractive product was obtained from a mixture of E10-16Ald and Z10-16Ald at a ratio of 100:8 [19]. Jung et al. (2000) revealed that E10-16Ald and Z10-16Ald at a ratio of 85:15 were detected in the pheromone gland of *C. punctiferalis*, and E10-16Ald and Z10-16Ald at ratios of 70:30–80:20 were the most efficient in males caught in Korea [20]. Additional research has identified several other female-produced unsaturated hydrocarbons, (*Z*)-9-heptacosene (Z9-27:HC) and (*Z*3,*Z*6,*Z*9)-3,6,9-tricosatrience (Z3,Z6,Z9-23:HC), as having potential pheromone activity of this species [21,22,23]. The research described below investigated the use of a 9:1 ratio of the singly unsaturated aldehydes, E10-16Ald and Z10-16Ald, in mating disruption experiments for *C. punctiferalis*.

In the present study, we conducted experiments over two years using different amounts of pheromone to demonstrate the disruption effect of the target species *C. punctiferalis.* The main objective of this study was to assess the effectiveness of MD as an alternative for controlling *C. punctiferalis*. Our study is the first to evaluate MD in chestnut orchards in Korea.

## 2. Materials and Methods

### 2.1. Experimental Sites and Experimental Design

Field trials were conducted over two years (2022–2023) in two commercial chestnut orchards located in central Korea, Orchard A (37°05′40″ N, 127°49′44″ E) and Orchard B (37°04′23″ N, 127°49′58″ E), in the Chungcheongbuk-do region. The two orchards were approximately 650 m apart (Figure S1A) and were both infested with *C. punctiferalis*. The trees were 20–40 years old and had a height of 10–15 m. The tree density was ca. 300 trees/ha. In 2022, two plots of ca. 1 ha each (experimental unit), separated by at least 50 m and 25 m, were selected in Orchard A and Orchard B, respectively. One of these units was chosen for the MD treatment, and the other was used as a control. MD dispensers were applied only once in April at an amount of 50 g/ha (TS) (3–5 MD pouch/tree) (Figure S1B,C). In 2023, three plots of ca. 1 ha each (experimental unit), separated by at least 50–70 m and 25–70 m, were selected in Orchard A and Orchard B, respectively. Two of these units were selected to receive the MD treatment, and the other was used as a control (Figure S1D,E). Two different treatments were applied as follows: MD dispensers were applied only once in April at an amount of 100 g/ha (one double application, TD) in one treatment plot and were applied twice in April and August at an amount of 50 g/ha (two-application treatment, TT) in the other treatment plot (Table 1). In Orchard A, natural organic insecticides against aphids were applied once in late July. The natural organic insecticides applied in Orchard A were a mixture of castor oil extract (30%), camellia seed extract (20%), and *Bacillus thuringiensis* culture fluid, while in Orchard B, herbicides and fungicides were applied to the plots.

### 2.2. Chemicals, MD Dispensers, and Monitoring Traps

MD was performed using the pheromone components (*E*)-10-hexadecenal (E10-16Ald) and (*Z*)-10-hexadecenal (Z10-16Ald) at a ratio of 9:1 and a pheromone dispenser consisting of halloysite beads impregnated with pheromones in a pouch (thermoplastic polyurethane film, 0.15 mm thickness, 25 × 70 mm) [24]. The pheromone mixture was prepared by one of our authors, following the methods by Vinczer et al. with a modification [25]. The MD dispenser was prepared as follows: The pheromone mixture was dissolved in hexane and soaked in the beads of halloysite (3 mm in diameter) for 6 h. After the hexane had evaporated, the halloysite beads impregnated with the pheromone mixture were sealed into the pouch. In each dispenser, 50 mg of the pheromone mixture and ca. 57 halloysite beads were combined. For MD, the MD dispenser (3–4 MD dispensers/tree) was hung on branches 2–3 m in height.

The population dynamics of *C. punctiferalis* were investigated using sex pheromone lures (E10-16Ald, Z10-16Ald, Z9-27:HC, Z2, Z6, and Z9-23:HC) and red delta traps (AD Corp., Andong, Republic of Korea). The pheromone lure was purchased from KIP, Inc. (Daejeon, Republic of Korea). Three traps per ha were hung on chestnut branches at 2–3 m in height on the outer surface of the tree canopy. The pheromone lures were replaced every four weeks, and the traps were inspected every week. At each inspection, the adhesive sheet was replaced with a new sheet. All the adhesive sheets were brought to the laboratory for counting, and all other nontarget moths were discarded.

### 2.3. Pheromone Residue in the MD Dispenser

To measure the amount of pheromone residue in the MD dispenser, the pouches of the MD dispenser, which contained 50 mg of the pheromone mixture, were placed in the chestnut orchard. The amount of pheromone in the pouch was extracted with hexane and measured by gas chromatography (GC) before hanging in the field, after 45 days and 90 days in 2022 and after 92 days (15 June–15 September 2023) in 2023. To extract the pheromone mixture, the halloysite beads from 3 MD dispensers were soaked in 100 mL of hexane for 10 h while stirring at room temperature. (*Z*)-8-Dodecenyl acetate was added to the extracts as an internal standard, and then GC was performed for quantitative analysis.

Quantitative analysis was carried out on an Agilent 6890 GC (Agilent Technologies, Santa Clara, CA, USA), in splitless mode with a flame ionization detector (FID). The samples were injected into an HP-1 column (15 m × 0.50 mm, 1.0 µm thickness, Agilent Technologies, Santa Clara, CA, USA). The oven temperature was programmed from 80 °C (2 min hold) to 250 °C at 7 °C/min, and to 280 °C at 5 °C/min, where the temperature was held for 15 min, then increased to 300 °C at 5 °C/min and held at the final temperature for 10 min. Nitrogen was used as the carrier gas at a rate of 2.0 mL/min. The inlet and detector were kept at 250 °C and 280 °C, respectively.

### 2.4. Evaluation of MCI and MD Effects

The male catch inhibition (MCI) for each year was calculated using the following equation: MCI (%) = 100 × (C − D)/C (where C is the number of captures in the control plots and D is the number of captures in the respective MD plots). The MD effect was evaluated using the following equation: MD efficacy (%) = 100 × (C′ − D′)/C′ (where C′ is the damage rate of the control plots and D′ is the damage rate of the respective MD plots). To calculate the damage rate, a sample of at least 1500 chestnut husks at each orchard was randomly hand-picked from the ground during the fruit fall in October. Nuts were visually inspected after collection for the presence of holes with frass. If there were holes with frass on the nuts, the nuts were cut, and the larvae of the insects (*C. punctiferalis, Cydia kurokoi* (Lepidoptera: Tortricidae) and *Curculio sikkimensis* (Coleoptera: Curculionidae) were investigated (Supplementary Figure S2). Only the nuts infested with *C. punctiferalis* larvae were counted for MD efficacy.

### 2.5. Data Analysis

The trap catch data were analyzed with a generalized linear mixed model (GLMM) with a Poisson distribution and log link function. A model was constructed using the number of males as the response variable, treatment (i.e., TS and control in 2022, and TD, TT), and week as explanatory variables, and location (i.e., Orchard A and B) as a random effect. The effects of the explanatory variables were tested using the type II likelihood ratio χ^2^ (LR) test. We also performed a post hoc Tukey’s test for pairwise comparisons among the treatments. These analyses were performed with the packages “lme4”, “car”, and “emmeans” in R software v. 4.3.3 [26]. The damaged chestnut fruits data were analyzed with the chi-squared test.

## 3. Results

### 3.1. Trap Captures

**Figure 1:**
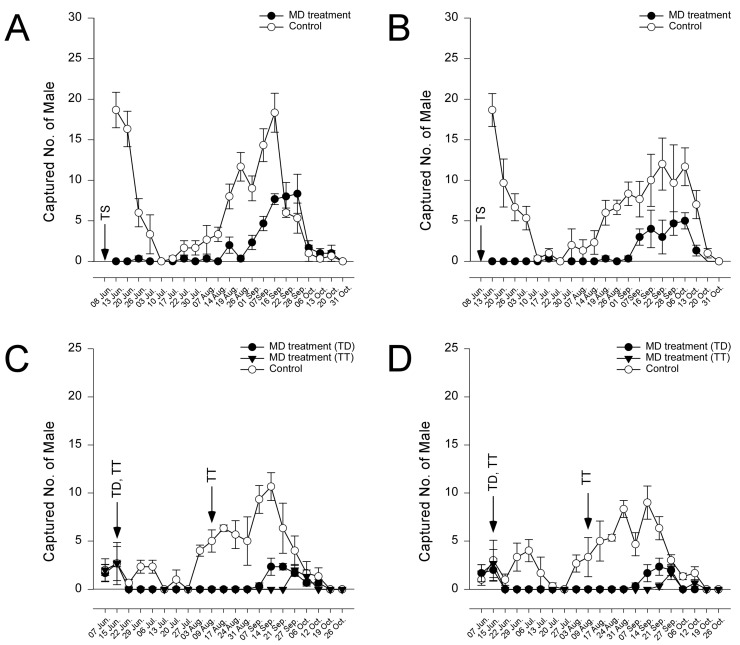
Number (mean ± SE) of male adults per week of *Conogethes punctiferalis* captured with pheromone-baited traps at all the surveyed sites. (**A**): Orchard A, 2022; (**B**): Orchard B, 2022; (**C**): Orchard A, 2023; (**D**): Orchard B, 2023. Arrows indicate the date of mating disruption. TS: 50 g/ha, TD: 100 g/ha, TT: 50 g/ha × 2.

The seasonal flight activity, reported as the number of male moths captured by traps for the sites, years, and MD and control plots, is presented in Figure 1. The number of moths exhibited a similar temporal pattern in both orchards. The first peaks occurred in mid-June, and the following peaks were observed in the middle to end of September 2022 in the control plot. In the MD treatment plot, the first peak observed in the control plot was not evident, but the second peak observed in the control plot was exhibited in the MD treatment plot. The number of captured males was affected by MD treatments in 2022 (*χ*^2^ = 306.97, *df* = 1, *p* < 0.0001) and in 2023 (*χ*^2^ = 342.04, *df* = 2, *p* < 0.0001), while the week had an effect on male captures in 2022 (*χ*^2^ = 5.28, *df* = 1, *p* < 0.021) but not in 2023 (*χ*^2^ = 0.12, *df* = 1, *p* = 0.7220). There was no significant difference between the TT and TD (pairwise comparison, *p* = 0.0972) in 2023. The MCI was 70.5% in Orchard A and 82.7% in Orchard B in 2022. In 2023, the MCIs in both the TD and TT MD treatment plots were 87.8% and 94.9%, respectively, in Orchard A and 89.6% and 95.1%, respectively, in Orchard B (Table 2).

### 3.2. Analyses of Pheromone Residues in Dispensers 

The residual pheromone content was calculated as a relative value, taking the amount of pheromone before treatment as 100%. The percentage of residual pheromone in halloysite was 38.82% after 45 days, 21.54% after 90 days in 2022, and 16.02% after 92 days in 2023. This percentage of residual pheromone was converted to the amount of pheromone per area (1 ha), yielding values of 19.41 g after 45 days and 10.77 g after 90 days in 2022, and 8.01 g after 92 days in 2023.

### 3.3. Chestnuts Damaged by Fruit-Feeding Pests

Throughout the experiments, *C. kurokoi* was the predominant pest, followed by *C. punctiferalis* and *C. sikkimensis.* These observations were consistent in both the MD treatment and control plots of Orchards A and B. The proportions of chestnuts infested by *C. kurokoi*, *C. punctiferalis,* and *C. sikkimensis* were 50.5–73.9%, 11.7–29.8%, and 11.0–29.3%, respectively.

### 3.4. Evaluation of the Efficacy of MD

The number of damaged chestnut fruits was affected by MD treatments in 2022 (*χ*^2^ = 126.85, *df* = 1, *p* < 0.0001) and in 2023 (*χ*^2^ = 143.39, *df* = 2, *p* < 0.0001). The MD efficacy (%) was calculated based on the total number of chestnut fruits collected and the number of fruits damaged by *C. punctiferalis*. In 2022, the MD efficacy was 63.9% in Orchard A, and 73.6% in Orchard B. In 2023, the MD efficacy by TD treatment and TT treatment was 60.2% and 77.9% in Orchard A and 50.9% and 64.8% in Orchard B, respectively (Table 3).

## 4. Discussion

Our study demonstrated the potential use of pheromone-based MD to reduce infestations by *C. punctiferalis* in chestnut orchards. In all the experiments, the MD treatment exhibited satisfactory MCI (70.5–82.7% in 2022, 87.8–95.1% in 2023) of males in the monitoring traps throughout the flight season, regardless of the type of orchard and total amount of MD pheromone used (Table 2). These results are consistent with the literature [27]. In addition, MD treatment showed good efficacy against *C. punctiferalis*, at a rate of 50.9–77.9% (Table 3). Although a dramatic decrease in trap catch was shown, a subsequent reduction in chestnut damage was not as great as expected. Similar results have been found in other studies on lepidopteran pests, for which a drastic decrease in the trap catch did not indicate effective suppression or reduced damage to the crop. The difference in the efficacies of MCI and MD could be attributed to the positioning of the monitoring traps and MD dispensers. The monitoring traps and MD dispensers were hung at a height of 2–3 m, which resulted in a high MCI efficacy due to the influence of the MD dispensers at this level. However, the flight activity of *C. punctiferalis* commonly occurs at heights of greater than 3 m, around the chestnut tree crowns. In these higher regions (>3 m), the influence of the MD dispensers could be reduced or negligible, allowing mating to occur and leading to increased damage. Although a strong reduction in the trap catch can be achieved at the height where the dispensers were placed, males may still find calling females further up in the trees if the pheromone does not disperse well enough to such heights. It is known that for the moth species infesting tall-growing trees, the capture of males can be higher in traps placed high up in the trees; therefore the efficacy of mating disruption could potentially reduce if the dispensers are placed at low heights [28]. In order to reduce the difference in the efficacy between MCI and MD, deploying the MD dispenser at a higher height would be needed. 

Generally, polyphagous species may be more challenging to control through MD because they can move to the treated area from other host plants such as oak and beech, in the case of chestnut tortrix moths [29,30,31,32]. *Conogethes punctiferalis* is a well-known polyphagous species [9,10,33,34,35]. At our experimental sites, the other host trees of *C. punctiferalis,* such as peach and apricot, were planted near the tested chestnut orchards. The mated females may migrate from adjacent untreated areas with different host plants into the MD-treated area, resulting in a moderate level of control efficacy despite the stronger MCI effect observed within the MD-treated area. Similarly, Svensson et al. (2018) suggested that a dramatic decrease in the trap catch did not correspond to a subsequent reduction in cone damage [28].

In MD treatments, the amount of pheromones is one of the main factors affecting success. To evaluate the proper amount and timing at which to deploy the MD dispenser, three different MD treatments were used in this experiment as follows: one dose of 50 g/ha (TS), one dose of 100 g/ha (TD), and two doses of 50 g/ha (TT). In the 2022 experiments, the number of male captures in treatment plots increased after August; thus, we deployed the MD dispenser at the TD level (100 g/ha) in the early season or deployed it under TT conditions to suppress mating in the tested area. Considering the MCI efficacy of MD after August 9, which was the TT deployment, TT had the greatest effect, followed by TD and TS deployment. The percentages of MD inhibition in TS were 53.7% and 74.1% in Orchards A and B, respectively. The percentages of MD inhibition in the TD treatment were 85.5% and 86.8% in Orchards A and B, respectively, and in the TT treatment, they were 94% and 94.4% in Orchards A and B, respectively. In August, the amount of residual pheromone in the MD-treated area in the TS treatment was expected to be 10–19 g, while in the TD and TT treatments, the amounts of residual pheromone in the MD-treated area were expected to be 20–40 g and 50 g, respectively. In August, it was observed that a pheromone amount exceeding 20 g/ha was necessary to achieve MD inhibition. While multiple MD treatments have been shown to be effective at inhibiting MD, cost considerations must also be accounted for.

Lepidopteran pheromones can be classified as Type I or Type II. Type I pheromones are characterized by unsaturated C10–C18 fatty alcohols, and their derivatives and type II pheromones are characterized by polyunsaturated hydrocarbons with C17–C25 straight chains and their corresponding epoxy derivatives [36]. The sex pheromone blend of *Dioryctria abietella* (Lepidoptera: Pyralidae), a pest of spruce seeds, was identified as (9*Z*,11*E*)-teradecadienyl acetate (Z9,E11-14OAc) (Type I) and (3*Z*,6*Z*,12*Z*,15*Z*)-pentacosapentaene (C25 pentaene) (Type II) [37,38]. Svensson et al. (2018) conducted MD experiments using both components, Z9,E11-14OAc and C25 pentaene, at a ratio of 1:10 and revealed that C25 pentaene had no disruption effect [28]. The pheromones of *C. punctiferalis* consisted of Type I (E10-16Ald and Z10-16Ald) and Type II (Z9-27:HC and Z9-27:HC, Z2,Z6,Z9-23:HC) pheromones. Considering a previous report, we conducted MD experiments using only the Type I pheromone components E10-16Ald and Z-10-16Ald. Mochizuki et al. (2002) reported the first commercial field example of resistance to MD in *Adoxophy honmai* (Yasuda) (Lepidoptera: Tortricidae) [39]. In this case, among the pheromone blends of *A. honmai*, only one major component was used for MD. In our study, among the pheromone components of *C. punctiferalis*, two Type I pheromones were used for MD. Consequently, the emergence of a resistant strain of *C. punctiferalis* is uncommon. However, early detection and ongoing monitoring are necessary to prevent such resistance.

Our experiments may provide the first pieces of information for using pheromones to control infestations of chestnut pests. Currently, the major pest of chestnut fruits in Korea is known as *C. punctiferalis* [9,35,40]. However, through this experiment, it was revealed that *C. kurokoi* was the most serious pest. *Cydia* species are commonly detected in chestnuts in Asia and Europe. However, there are few descriptions of *Cydia* species as pests of chestnut in Asia. Choo et al. (2001) reported that the ratio of *C. kurokoi*, *C. punctiferalis*, and *C. sikkimensis* in the damaged chestnuts was 27.7%, 40.7%, and 31.5%, respectively, in 1999 [41]. Komai and Ishikawa (1987) reported that in Japan, *C. glandicolana* and *C. kurokoi* were detected in chestnuts imported from China [42]. However, the damage caused by *C. glandicolana* and *C. kurokoi* has not been reported. Unlike in Asia, much more information on the *Cydia* species as pests of chestnuts has been reported in Europe. *C. fagiglandana* and *C. splendana* are known as key pests, and a pheromone trap has been used for monitoring in Italy [30,43,44]. An attempt to control these two species via pheromone-based mating disruption has also been reported [27]. To understand the pest status in chestnut orchards in Korea, further investigations are needed.

## 5. Conclusions

In this study, we demonstrated the efficacy of pheromone-based mating disruption in reducing infestations caused by *C. punctiferalis* from chestnut orchards and showed consistent male catch inhibition and management efficiency. Although damage was reduced, it was not as much as expected in MCI data, likely due to the ability of this species to move from other host plants. The amount and timing of pheromone deployment affect the efficacy of mating inhibition, considering the cost and potential development of resistance. Additionally, the experiment revealed that *C. kurokoi* is a significant pest of chestnut, highlighting the need for further investigation into its pest status in Korean chestnut orchards.

## Figures and Tables

**Table 1:** Treatments tested in the different years in chestnut orchards.

Year	Site	Treatment	Area (ha)	Treatments (Amount of MD)
2022	Orchard A	MD (TS)	1.07	54 g
		Control	0.94	-
	Orchard B	MD (TS)	0.86	48 g
		Control	0.92	-
2023	Orchard A	MD (TD)	1.02	102 g
		MD (TT)	0.98	49 g × 2
		Control	0.94	-
	Orchard B	MD (TD)	0.92	92 g
		MD (TT)	0.86	43 g × 2
		Control	1.14	-

MD (TS): Mating disruption (MD) dispensers applied only in April at 50 g/ha; MD (TD): MD dispenser applied only in April at 100 g/ha; MD (TT): MD dispenser applied twice in April and August at 50 g/h.

**Table 2:** Male catch inhibition (MCI, %) of *Conogethes punctiferalis* by mating disruption treatment.

Site	2022			2023		
	Treatment	Total No. Captured *C. punctiferalis*	Male Catch Inhibition (MCI, %)	Treatment	Total No. Captured *C. punctiferalis*	Male Catch Inhibition
Orchard A	MD (TS)	114	70.5	MD (TD)	24	87.8
	Control	386	-	MD (TT)	10	94.9
	-	-	-	Control	197	-
Orchard B	MD (TS)	66	82.7	MD (TD)	19	89.6
	Control	382	-	MD (TT)	9	95.1
	-	-	-	Control	183	-

MD (TS): Mating disruption (MD) dispensers applied only in April at 50 g/ha; MD (TD): MD dispenser applied only in April at 100 g/ha; MD (TT): MD dispenser applied twice in April and August at 50 g/h.

**Table 3:** Chestnut fruits damaged by *Conogethes punctiferalis* and efficacy of mating disruption (MD).

**Year: 2022**				
**Site**	**Treatment**	**Total No. Chestnut Fruit Collected**	**No. Damaged Chestnut Fruit (%)**	**MD Efficacy (%)**
Orchard A	MD (TS)	1067	86 (8.06)	63.9
	Control	908	203 (22.35)	-
Orchard B	MD (TS)	787	52 (6.61)	73.6
	Control	783	196 (25.03)	-
**Year: 2023**				
Orchard A	MD (TD)	1083	75 (6.92)	60.2
	MD (TT)	1171	45 (3.84)	77.9
	Control	908	158 (17.40)	-
Orchard B	MD (TD)	1064	89 (8.36)	50.9
	MD (TT)	1134	68 (6.00)	64.8
	Control	1097	187 (17.04)	-

MD (TS): Mating disruption (MD) dispensers applied only in April at 50 g/ha; MD (TD): MD dispenser applied only in April at 100 g/ha; MD (TT): MD dispenser applied twice in April and August at 50 g/h.

## Data Availability

The data presented in this study are available on request from the corresponding authors.

## Supplementary Materials

The following supporting information can be downloaded at: https://www.mdpi.com/article/10.3390/insects15060445/s1, Figure S1: The location of Orchard A and Orchard B (A) and the area of treatment in Orchard A and B; B: Orchard A in 2022, C: Orchard B in 2022, D: Orchard A in 2023, E: Orchard B in 2023. B, C: Red line indicating the single-dose treatment (TS, 50 g/ha) area. Blue line indicating the control area. D, E: Orange line indicating the two-application treatment (TT, 50 g/ha in June and August) area, purple line indicating the double-dose treatment (TD, 100 g/ha) area, and blue line indicating the control area; Figure S2: The larvae in the damaged chestnut fruit.
