# Supplementary material for: Pheromone-Based Mating Disruption of Conogethes punctiferalis (Lepidoptera: Crambidae) in Chestnut Orchards
Source: Insects. 2024 Jun 12;15(6):445. doi: 10.3390/insects15060445 (PMC11203852; doi:10.3390/insects15060445)
Supplement: Supplementary file 1 [file insects-15-00445-s001.zip › insects-3012156-supplementary.pdf]

Supplementary Materials

Pheromone-based mating disruption of *Conogethes punctiferalis* (Lepidoptera: Crambidae) in chestnut orchards

Junheon Kim[1,*,†] Seongchae Jung[2,*], Young Un Kim[2]

[1]Forest Entomology and Pathology Division, National Institute of Forest Science, Seoul, 02455, Republic of Korea

[2]AD Corporation, Andong 36729, Republic of Korea

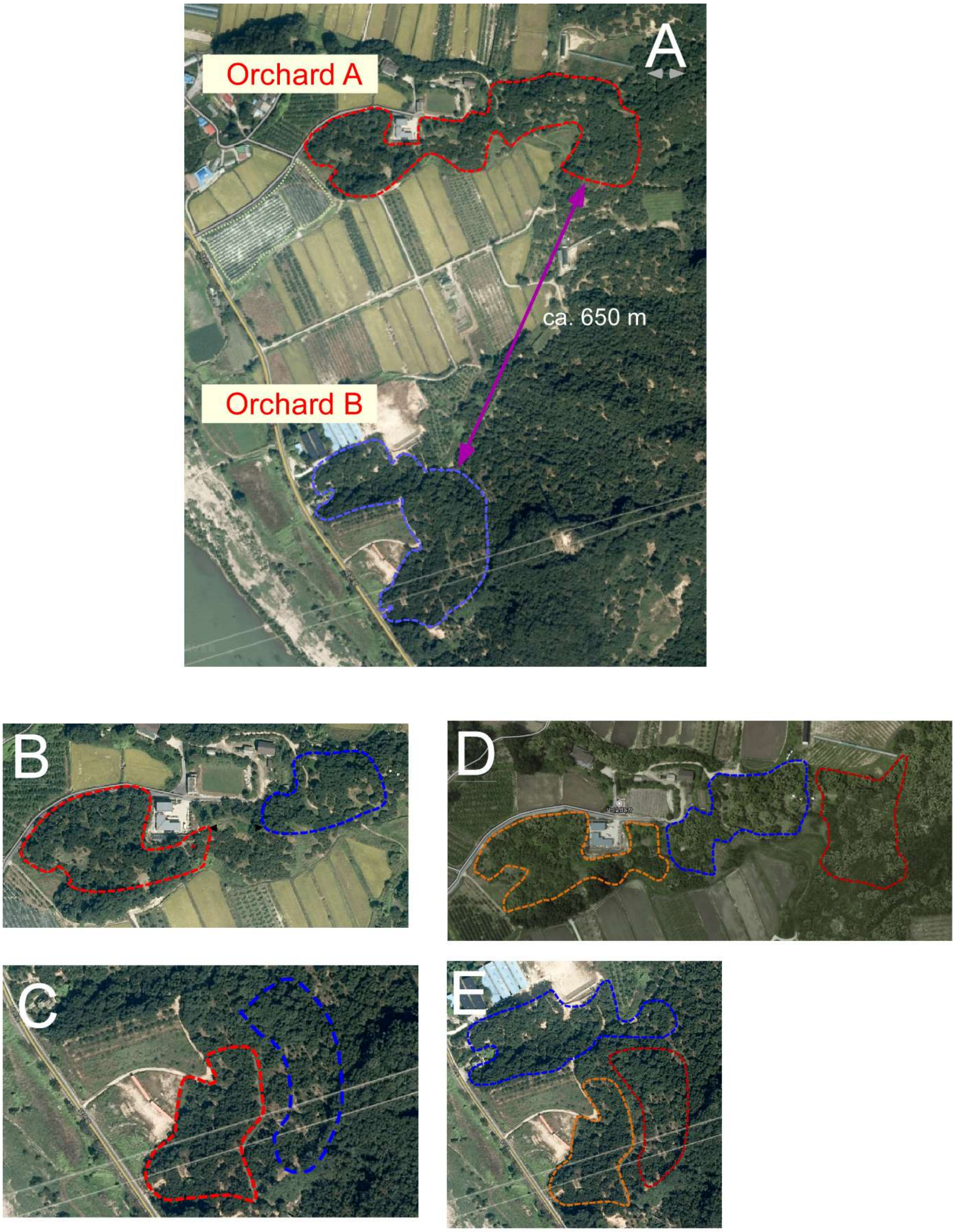

Figure S1. The location of Orchard A and Orchard B (A) and the area of treatment in Orchard A and B; B: Orchard A in 2022, C: Orchard B in 2022, D: Orchard A in 2023, E: Orchard B in 2023. B, C: Red line indicating the single-dose treatment (TS, 50 g/ha) area. Blue line

indicating the control area. D, E: Orange line indicating the two-application treatment (TT, 50 g/ha in June and August) area, purple line indicating the double-dose treatment (TD, 100 g/ha) area, and blue line indicating the control area.

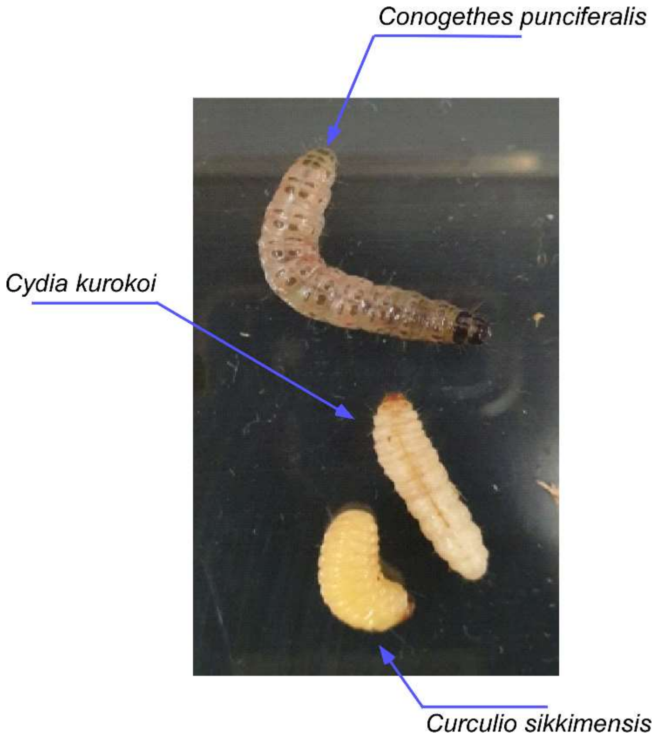

Figure S2. The larvae in the damaged chestnut fruit.
